# Correction: Effects of trap confinement on personality measurements in two terrestrial rodents

**DOI:** 10.1371/journal.pone.0229220

**Published:** 2020-02-13

**Authors:** Allison M. Brehm, Sara Tironi, Alessio Mortelliti

## Notice of Republication

This article was republished on January 30, 2020, to remove an erroneous sentence from the Introduction and to correct the Academic Editor’s affiliation. The publisher apologizes for these errors. Please download this article again to view the correct version. The originally published, uncorrected article and the republished, corrected articles are provided here for reference.

## Supporting information

S1 File Originally published, uncorrected article. (PDF)

S2 File Republished, corrected article. (PDF)

## References

[1] Brehm AM, Tironi S, Mortelliti A (2020) Effects of trap confinement on personality measurements in two terrestrial rodents. PLoS ONE 15(1): e0221136 10.1371/journal.pone.0221136 31986141 PMC6984697

