# Supplementary material for: Effects of trap confinement on personality measurements in two terrestrial rodents
Source: PLoS One. 2020 Jan 27;15(1):e0221136. doi: 10.1371/journal.pone.0221136 (PMC6984697; doi:10.1371/journal.pone.0221136)
Supplement: S1 Table — (DOCX) [file pone.0221136.s001.docx]

**Supporting information**

| **S1 Table. Repeatability estimates for target behaviors measured in three behavioral tests (handling bag, emergence, and open-field) in deer mice (*Peromyscus maniculatus*) and southern red-backed voles (*Myodes gapperi*).** | | | | | | | |
| --- | --- | --- | --- | --- | --- | --- | --- |
|  | Behavioral Variable | Mean | Range | Repeatability (95% CI) | Observations | Individuals |  |
|  | ***P. maniculatus*** |  |  |  |  |  |  |
|  | Handling time | 15.41 | (0, 60) | **0.836 (0.807, 0.862)** | 1122 | 376 |  |
|  | Latency to emerge | 27.17 | (0, 180) | **0.812 (0.780, 0.842)** | 1122 | 376 |  |
|  | Time at end of tunnel | 7.15 | (0, 180) | **0.863 (0.841, 0.884)** | 1122 | 376 |  |
|  | Mean speed (m/sec) | 0.10 | (0, 0.25) | **0.832 (0.809, 0.853)** | 1791 | 603 |  |
|  | Prop. time grooming | 0.11 | (0, 0.96) | **0.762 (0.735, 0.792)** | 1791 | 603 |  |
|  | Rear rate | 0.19 | (0, 0.68) | **0.809 (0.785, 0.831)** | 1791 | 603 |  |
|  | Prop. time center | 0.03 | (0, 0.73) | **0.775 (0.747, 0.804)** | 1791 | 603 |  |
|  | ***M. gapperi*** |  |  |  |  |  |  |
|  | Handling time | 47.77 | (0, 60) | **0.675 (0.62, 0.726)** | 940 | 305 |  |
|  | Latency to emerge | 34.94 | (0, 180) | **0.831 (0.799, 0.859)** | 940 | 305 |  |
|  | Time at end of tunnel | 12.05 | (0, 180) | **0.823 (0.791, 0.851)** | 940 | 305 |  |
|  | Mean speed (m/sec) | 0.05 | (0, 0.20) | **0.792 (0.765, 0.818)** | 1558 | 529 |  |
|  | Prop. time grooming | 0.06 | (0, 0.81) | **0.729 (0.694, 0.764)** | 1558 | 529 |  |
|  | Rear rate | 0.09 | (0, 0.56) | **0.770 (0.739, 0.801)** | 1558 | 529 |  |
|  | Prop. time center | 0.04 | (0, 0.99) | **0.827 (0.805, 0.850)** | 1558 | 529 |  |
| Repeatability was calculated from univariate mixed-effect models with identity included as a random effect. Parametric bootstrapping was used to calculate 95% confidence intervals. See Methods for more information. Significant repeatability estimates are shown in bold. | | | | | | | |
