# Supplementary material for: Correction: Effects of trap confinement on personality measurements in two terrestrial rodents
Source: PLoS One. 2020 Feb 13;15(2):e0229220. doi: 10.1371/journal.pone.0229220 (PMC7018005; doi:10.1371/journal.pone.0229220)
Supplement: S2 File — (PDF) [file pone.0229220.s002.pdf]

PLOS ONE

RESEARCH ARTICLE

Effects of trap confinement on personality measurements in two terrestrial rodents

Allison M. Brehm, Sara Tironi, Alessio Mortelliti*

Department of Wildlife, Fisheries, and Conservation Biology, University of Maine, Orono, ME, United States of America

* alessio.mortelliti@maine.edu

Check for updates

OPEN ACCESS

Citation: Brehm AM, Tironi S, Mortelliti A (2020) Effects of trap confinement on personality measurements in two terrestrial rodents. PLoS ONE 15(1): e0221136. https://doi.org/10.1371/journal.pone.0221136

Editor: Julien Martin, University of Ottawa, Canada

Received: July 24, 2019

Accepted: December 17, 2019

Published: January 27, 2020

Copyright: © 2020 Brehm et al. This is an open access article distributed under the terms of the Creative Commons Attribution License, which permits unrestricted use, distribution, and reproduction in any medium, provided the original author and source are credited.

Data Availability Statement: All original data files will be available from the Figshare database 10.6084/m9.figshare.11418405.

Funding: AM received funds for McIntire Stennis project numbers ME041620 and ME041913 through the Maine Agricultural & Forest Experiment Station supported by the USDA National Institute of Food and Agriculture (https://nifa.usda.gov/program/mcintire-stennis-capacity-grant), AM received funding from the the University of Maine RRF Graduate Assistantship and RRF Undergraduate Assistantship, AMB received funds from the American Society of Mammalogists

Abstract

In recent years, consistent individual differences in behavior, or personalities, have been a topic of increasing interest as researchers strive to understand and predict the responses of individuals and populations to anthropogenic changes. Behavioral studies in wild populations often require that animals are live trapped before behavioral observation can occur, and this is especially true in studies investigating animal personalities. However, it is unknown whether the amount of time confined to a live trap may regulate the behavior of trapped individuals. Specifically, if the duration of trap confinement directly influences behavior, then by obtaining wild animals through live trapping we may be confounding the very measurements of greatest interest. To investigate whether the duration of trap confinement influences the behavior of trapped individuals, we performed a study on two small mammal species, focusing specifically on personality traits. We positioned high-definition trail cameras facing Longworth small mammal traps in the field to observe capture events and record the time of capture. We then measured personality in captured deer mice (*Peromyscus maniculatus*) and southern red-backed voles (*Myodes gapperi*) using three standardized tests, and through linear and generalized linear models we found that the time an animal had spent confined to a trap before testing did not affect 86% of behaviors exhibited. Our results showed two weak behavioral effects of confinement duration on boldness and docility resulting from an interaction between the duration of confinement and whether or not an individual was naïve to trapping. Our results suggest that behavioral measurements of wild, trapped small mammals are not determined by the time spent confined to a trap. However, researchers should use caution and consider whether an animal is naïve to trapping during analysis since habituation to the live trap may play a role in the effects of confinement duration on behavior.

Introduction

Over the past few decades, the acknowledgment that many taxa display consistent individual differences in behavior, or *personalities*, has become widespread [1–4]. Personalities are heritable [5], have consequences for fitness [6–9], and can limit the ability of individuals to exhibit

(Grants in aid of Research) and AM received funds from the Penobscot Experimental Forest Research Funds. The funders had no role in study design, data collection and analysis, decision to publish, or preparation of the manuscript.

Competing interests: The authors have declared that no competing interests exist.

behavioral plasticity [10]. This can result in trade-offs where certain personality types perform well in some ecological contexts but not in others [11]. Because individual personalities can determine the response of individuals to changing environments [12,13] and have important ecological implications [14–16], personality studies in wild populations will likely continue to increase as researchers strive to understand and predict the responses of individuals and populations to anthropogenic changes [17–20].

Studies of personality in wild populations usually require that wild animals are live trapped so that one or more standardized behavioral tests can be undertaken, but see [21–24] for methods of personality observation in non-captured animals. Because live trapping may induce stress [25–30], the process of capturing animals and subsequently measuring their personality offers additional challenges. Specifically, the stress of being trapped might influence the behaviors exhibited by wild animals. When trap-induced stress is unequal among individuals or among capture events and cannot be controlled for during analyses, this could confound the very behaviors at the core of the research.

Several studies have explored the relationship between live trapping and the stress response of animals [28–30]. It is generally accepted that the stress of being captured activates the sympathetic nervous system (secreting catecholamines) as well as the hypothalamic-pituitary-adrenocortical (HPA) axis (releasing glucocorticoids into the bloodstream) [28,31]. The hormones secreted from the sympathetic nervous system during the stress response can elevate breathing rate, heart rate, and blood pressure [28] which, following exposure to a threat (such as a predator attack), stimulates the mobilization of energy to facilitate an escape. Alternatively, the glucocorticoids released from the HPA axis can suppress digestion, inflammation, and reproduction [31]. When an animal is confined to a trap, this prolonged stressor may result in higher concentrations of stress-related hormones like catecholamines and glucocorticoids after longer durations spent in a trap [29], perhaps impacting behaviors exhibited during routine behavioral tests such as grooming and time spent moving [32–34]. Thus far, studies looking to assess this phenomenon have focused on the hormonal/physiological response to trap-induced stress and results have been mixed [28,30,35]. For example, live trapping does induce an initial stress response (measured using fecal glucocorticoid levels and corticosterone concentrations) in southern red-backed voles (*Myodes gapperi*) and meadow voles (*Microtus pennsylvanicus*), but longer times spent in traps do not correlate with increased stress-related hormone levels [28,35]. By contrast, studies found that in deer mice (*Peromyscus maniculatus*) and American red squirrels (*Tamiasciurus hudsonicus*) prolonged time spent in traps was positively correlated with stress-related hormone levels [30,35]. In either scenario, it is unknown whether the time spent in traps may affect behavioral responses, since a change in stress-related hormones does not necessarily precede a change in behavior.

If confinement duration affects behaviors exhibited during routine testing, this could result in misinterpretation of results and may mask the presence of repeatable behavioral traits in populations of interest. For example, if an individual is captured twice and its behavior assessed each time, but the individual spends one hour confined to a trap on the first capture and eight hours confined to a trap on the second capture, the difference in confinement duration may obscure any consistency in this individual's observed behaviors. Alternatively, if an individual's personality influences how quickly it enters a trap, meaning that the boldest individuals enter traps earlier (experiencing longer durations of confinement) this could lead to increased stress levels in only the boldest individuals. If the heightened stress levels caused a behavioral change, for example by causing individuals to behave in a shyer manner, truly bold individuals would appear to act similarly to truly shy individuals, but only because they have been confined to traps longer. This type of confoundment would require studies using

behavioral data from trapped animals to further investigate the minimum duration of confinement that alters the behavioral response, and then control for confinement duration. This could be done by: checking traps more frequently, recording the time of capture (obtained using videos from camera traps placed on live traps) then controlling for the duration using imposed covariates in analysis, or using devices that signal when a capture has been made so that animals can be removed promptly [36,37]. Empirical evidence is needed to explore the relationship between the time spent in a trap and the behavioral response.

The objective of this study was to assess whether personality measurements obtained from live trapped individuals are being confounded by the amount of time spent inside of a trap. Specifically, we sought to determine whether confinement duration affects the behaviors exhibited in routine behavioral tests. To meet this objective, we conducted a field experiment focused on the deer mouse and the southern red-backed vole in study populations that have been the subject of previous personality research by the authors [16,38]. Using high-definition trail cameras positioned facing Longworth small mammal traps in the field, we quantified the duration of time that individuals had spent inside a trap before standardized behavioral tests were performed the following morning. Using these data, we evaluated whether behaviors exhibited in behavioral tests varied with the time spent inside the trap.

Results from this study will have implications for researchers who measure behavioral traits following the live-capture of an animal. These results will highlight whether we should take additional steps to ensure that our behavioral measurements are accurate and not unduly influenced by the time spent in the trap.

Materials and methods

Study site and small mammal trapping

This study was conducted in the Penobscot Experimental Forest (PEF, 44° 51' N, 68° 37' W) at the southern edge of the Acadian forest in east-central Maine, USA. This experimental forest consists of forest units chosen at random and logged separately with varying silvicultural treatments (minimum of two replicates per treatment). Management units average 8.5 ha in area (range 8.1–16.2 ha) and nearly 25 ha of forest (retained in two separate units) serves as reference and has remained unmanaged since the late 1800s [38,39].

We implemented a large-scale mark-recapture study on six trapping grids (Fig 1): two control (located in reference forest) and four experimental (two replicates in even-aged forest units and two in units treated with a two-stage shelterwood with reserves). Trapping grids were 0.81 ha in area and consisted of 100-flagged points spaced 10 m apart. We positioned trapping grids close to the center of the management unit to minimize edge effects (mean distance between grids was 1.44 km; greater than the movements of our study species). We positioned one Longworth trap at each flagged point. Traps were bedded with cotton and baited with a mixture of sunflower seeds, oats, and freeze-dried mealworms. We trapped at each trapping grid for three consecutive days and nights and checked traps each morning and evening. Trapping occurred once per month for five consecutive months each year (June–October 2016, 2017, 2018).

Behavioral tests

We used three standard behavioral tests to measure personality of trapped individuals: an *emergence* test to assess boldness [32,40], an *open-field* test to measure activity and exploration in a novel environment [41,42], and a *handling bag* test to measure docility and the response to handling by an observer [43–46]. We performed behavioral tests in the order above prior to handling or marking. All tests and processing occurred at a base area in the home grid of the

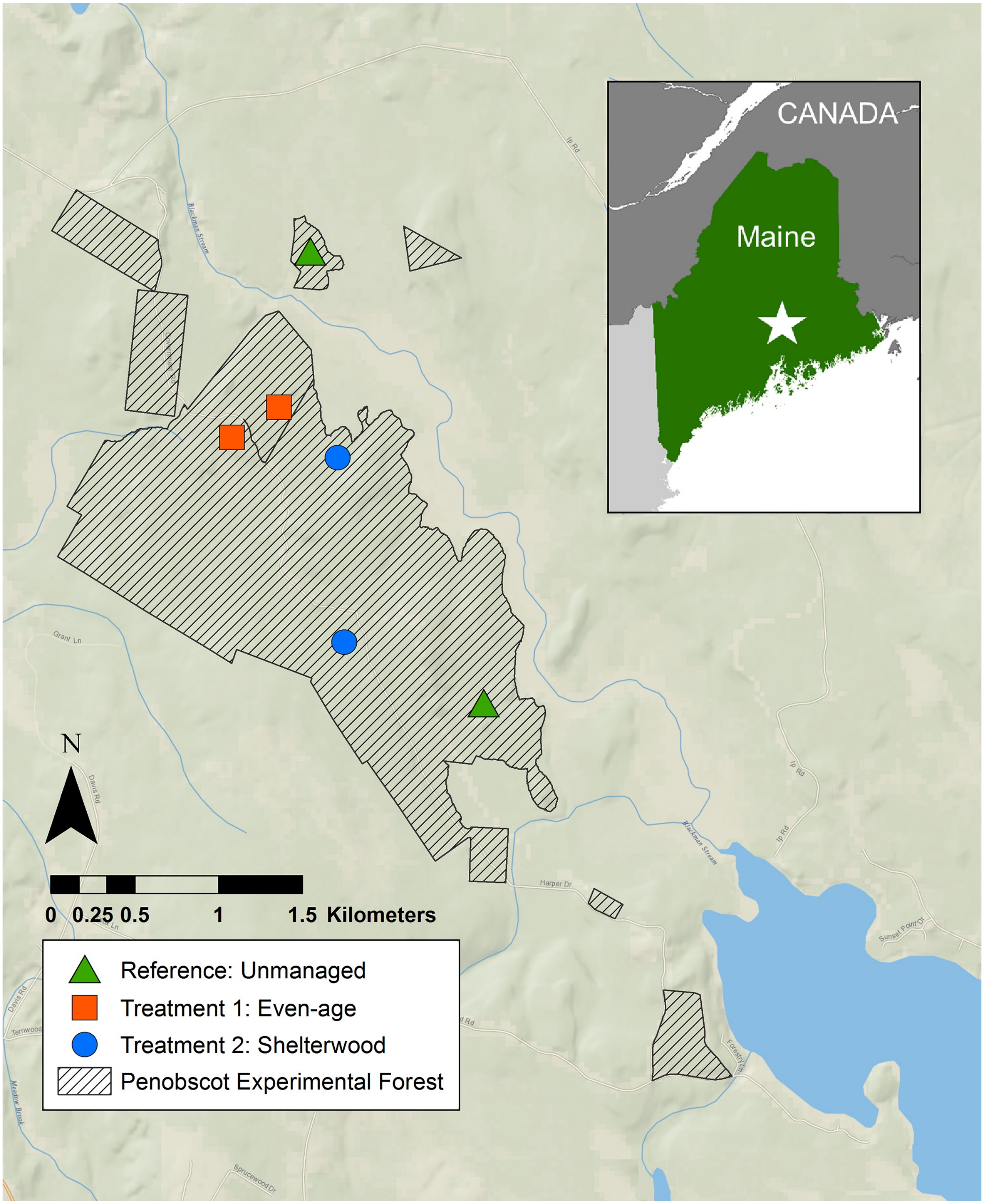

Fig 1. Map of the study area at the Penobscot Experimental Forest, Maine U.S.A. (PEF, 44˚ 51' N, 68˚ 37' W).

https://doi.org/10.1371/journal.pone.0221136.g001

focal individual. For detailed information about how behavioral tests were performed in the field, see methods described by Brehm *et al.* (2019).

After the completion of the behavioral tests, we recorded sex, body mass (measured using a 100 g Pesola Lightline spring scale), reproductive status (classed as either reproductively active or not based on the presence of an enlarged scrotum, perforated vagina, or signs of pregnancy or lactation), and age class (juvenile or adult). New individuals were anesthetized using isoflurane and tagged with PIT tags (Biomark MiniHPT8) subcutaneously at the mid back. Animals were also marked with a small animal ear tag (National Band, Style 1005–1) and a distinctive haircut. Haircuts were given using one or a combination of small cuts on the following locations: left shoulder, right shoulder, left mid-back, right mid-back, left rear, right rear. These cuts allow for visual identification in camera traps and are superior to methods using dye because they will show up in black and white photo and video. Once per month, we measured the body and tail length of captured individuals (while under anesthesia), and we released all individuals at the exact site of capture post-processing.

To quantify behavior from videotaped emergence and open-field tests, recordings were played back in the laboratory. For emergence tests, an observer recorded the following: the latency for the animal to emerge (defined as all four feet having left the trap), and the total time spent at the end of the tunnel before emerging. It was determined that an animal was at the end of the tunnel if its nose protruded from the tunnel opening. Open-field tests were analyzed using the behavioral tracking software ANY-maze © (version 5.1; Stoelting CO, USA) to record each individual's mean speed, distance traveled, relative location in the arena, and supplemental behaviors were recorded like grooming, rearing, and jumping. For the remainder of analyses, we focused on a reduced number of non-redundant and repeatable behavioral variables. See Brehm *et al.* (2019) for a complete list and biological interpretation of the behaviors used in this study system.

Monitoring capture events

To observe the event of an individual's capture and calculate the time spent inside the trap before behavioral testing, we positioned camera traps (Bushnell NatureView HD 119740) facing the door of the Longworth trap and its surroundings. We monitored Longworth capture events using camera traps from July–October 2018 (936 total camera trap nights). Cameras were positioned ~50–100 cm from the trap at a height of ~50 cm. Thirteen camera traps were used in total and were positioned on a subset of the 100 available trap locations (Fig 2). We chose camera locations to optimize the chance of observing capture events (hence, we chose trap locations that had successful captures during the previous month). Cameras were positioned simultaneously with Longworth traps and were kept active for the same duration as the traps (three consecutive days and nights at each study grid). We programmed cameras to record a one-minute video whenever movement was perceived (with a one-second delay between videos). Because camera traps occasionally fail to detect movement, we also programmed them to take a one-minute video once per hour (the "field scan" setting). This allowed us to approximate the hour of capture in an instance where the camera failed to trigger at the capture event.

Videos of capture events were played back in the laboratory, and an observer identified the individual by pairing the information of the date and trap with available capture data. The observer then recorded the time that the individual entered the trap and calculated the total time (in minutes) spent inside the trap before behavioral testing (taken from the time stamp of the open-field video for consistency). This variable will be referred to hereafter as "time in trap". See S1 Video and S2 Video in the supporting information for examples of observed capture events.

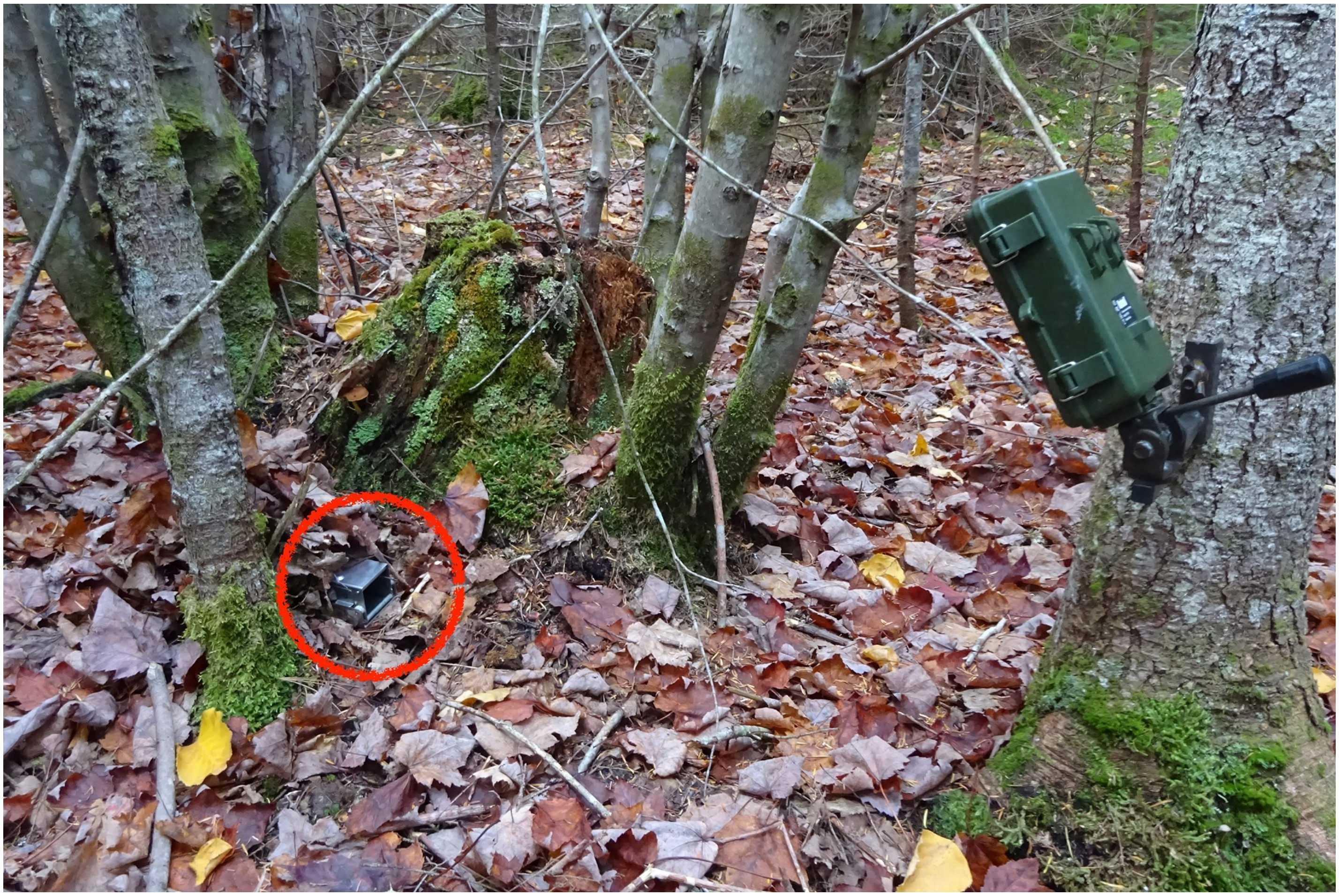

Fig 2. A camera trap (Bushnell NatureView HD) monitors a Longworth trap in the field (circled in red).

https://doi.org/10.1371/journal.pone.0221136.g002

Data analysis

To determine which behaviors could be considered personality, we first performed a repeatability analysis on the behavioral variables obtained from the emergence, open-field, and handling bag tests [47,48]. For this analysis, we used data from our study population collected during the 2016, 2017, and 2018 field seasons and used methods described in detail by [16,38].

Once it was determined which behaviors were repeatable and could be considered personality, we sought to determine whether these behaviors would be influenced by the time spent inside the Longworth trap before behavioral testing. We used a nested hypothesis testing approach [49] using linear models and generalized linear models with the repeatable behaviors as response variables. We used only the individual's behavioral measurement on the specific occasion when its capture was recorded on a camera trap. In the instances where we had repeated measures from the same individual (because we recorded their capture on a camera trap in subsequent trapping sessions– 18 out of 92 individuals), we used only the first event. This allowed us to avoid using mixed-effects models for only a few cases where measures were repeated [50]. Proportional response variables were logit-transformed to meet the assumptions of normality, and count variables were examined using generalized linear models with a poisson or negative binomial family (depending on dispersion).

We introduced predictor variables one by one to build a base model to control for most of the variability in the data. Continuous predictor variables were z-standardized. Predictor variables included sex, body condition, silvicultural treatment, trapping session, body mass, and a variable termed "naïve" which controlled for whether the animal had been captured previously or was naïve to trapping. Models containing each of these variables alone were compared to the null model using the Akaike information criterion corrected for small sample size (AICc) [49,51] and models within 2.0 ΔAICc of the top model were considered to have equal support. If more than one model scored better than the null, we tested a model including multiple additive effects. Once this base model was built, we compared this model to the same model with the addition of the variable "time in trap" to see whether this addition improved the model by AICc. Previous research has shown that males and females may respond differently to trap-induced stress [30], so we subsequently tested for an interaction between the time spent in the trap and sex. Last, to determine whether individuals who are naïve to trapping may be impacted by the time spent inside the trap differently than individuals who have been captured previously, we tested for an interaction between time spent in the trap and the variable "naïve".

Ethical note

Animal trapping, handling, and marking procedures were approved by the University of Maine's Institutional Animal Care and Use Committee (IACUC number A2015_11_02). Animals were anaesthetized with isoflurane prior to tagging, and tagging equipment was sanitized with 70% isopropyl alcohol in between animals. All small mammal handling was performed by trained researchers, and all efforts were made to minimize animal stress during the procedure.

Results

Repeatability analysis

We examined behavioral data collected over three trapping years in our study population from standardized tests for 1791 observations from 603 individual deer mice and 1558 observations from 529 individual red-backed voles. The mean number of repeated observations per individual was 1.7 ± 1.02 (range: 1–6) for deer mice and 1.6 ± 0.84 (range: 1–5) for red-backed voles. We selected seven significantly repeatable, non-redundant behavioral variables, with a mean repeatability value of 0.81 (95% CI: 0.79, 0.84) for deer mice and 0.78 (95% CI: 0.74, 0.81) for voles (S1 Table). These highly repeatable behaviors can be considered personality in our study populations [52,53]. The number of observations and individuals shown in S1 Table differ for behavioral variables obtained from the emergence and handling bag tests since these tests were not performed in 2016.

Trap confinement analysis

The mean time confined to a trap (in minutes) was 611 ± 218 (range: 74, 1085). This dataset included the capture events from 46 individual deer mice and 43 individual red backed voles for which we performed behavioral tests on the same occasion that a capture was recorded. In 12 out of 14 top models (~86%) predicting behaviors exhibited in standardized tests, the top model did not include "time in trap". Instead, out of the predictor variables considered (sex, body condition, silvicultural treatment, trapping session, body mass, and the variable "naïve") behaviors in deer mice were predicted by trapping session and body mass (Table 1). Deer mice with greater body mass showed longer latencies to emerge from the emergence test and the proportion of time spent grooming in the open-field test correlated positively with trapping

Table 1. Model output of top-ranked linear models* predicting behaviors performed during standardized tests in deer mice (*Peromyscus maniculatus*) and southern red-backed voles (*Myodes gapperi*).

P. maniculatus							
**Latency to emerge**	**β**	**St.Error**	**P-value**	**Prop. time grooming**	**β**	**St.Error**	**P-value**
(Intercept)	1.21	0.08	<0.001	(Intercept)	-3.88	0.51	<0.001
Body mass	0.26	0.08	0.003	Session	0.58	0.16	<0.001
R-squared	0.20			R-squared	0.23		
Observations	41			Observations	46		
**Prop. time center**	β	St.Error	P-value				
(Intercept)	-3.52	0.123	<0.001				
Time in trap	0.17	0.12	0.18				
Naïve	0.04	0.17	0.82				
Time in trap*Naïve	-0.53	0.17	0.005				
R^2	0.19						
Observations	46						
M. gapperi							
**Handling time**	β	St.Error	P-value				
(Intercept)	45.37	3.68	<0.001				
Time in trap	-12.4	3.71	0.002				
Naïve	6.04	4.53	0.19				
Time in trap*Naïve	11.3	4.71	0.02				
R^2	0.28						
Observations	43						

* Only results from the top model (based on AICc scores) are shown. We have omitted occasions where the null model was the top model. See materials and methods for more information.

https://doi.org/10.1371/journal.pone.0221136.t001

session. In two cases, (once for deer mice and once for voles) the top model included an interaction between "time in trap" and whether or not the individual was naïve to trapping (Fig 3A and 3B). Model fit was relatively low for top models (excluding those where the top model included only an intercept), with an average multiple R-squared value (R^2) of 0.23 (Table 1).

Discussion

Though previous research has suggested that live trapping may produce a stress response in small mammals, our study finds that prolonged confinement to a live trap does not seem to alter behavior in the deer mouse and the southern red-backed vole. In an experiment wherein we studied the effects of trap confinement on repeatable behavioral variables, our major finding was that for these species, 12 out of 14 behaviors exhibited during routine behavioral tests were not affected by the amount of time that individuals had spent confined in traps. In the two instances where the time spent confined in traps did predict behavior, effect sizes were relatively small, and the direction of the relationship was different for individuals who were naïve to trapping than those who had been trapped previously, indicating that an individual's previous experience with a trap can influence whether or not trap confinement impacts behavior. Overall, these results suggest that behavioral data collected from wild, trapped small mammals is not confounded by the trapping process and, where an effect might be present, the predictive power of the time spent confined to traps is relatively weak and possibly not affecting the overall interpretation of results.

Although previous research has not explored the effects of live trapping on personality measurements specifically, studies have investigated the impacts of live trapping on hormonal

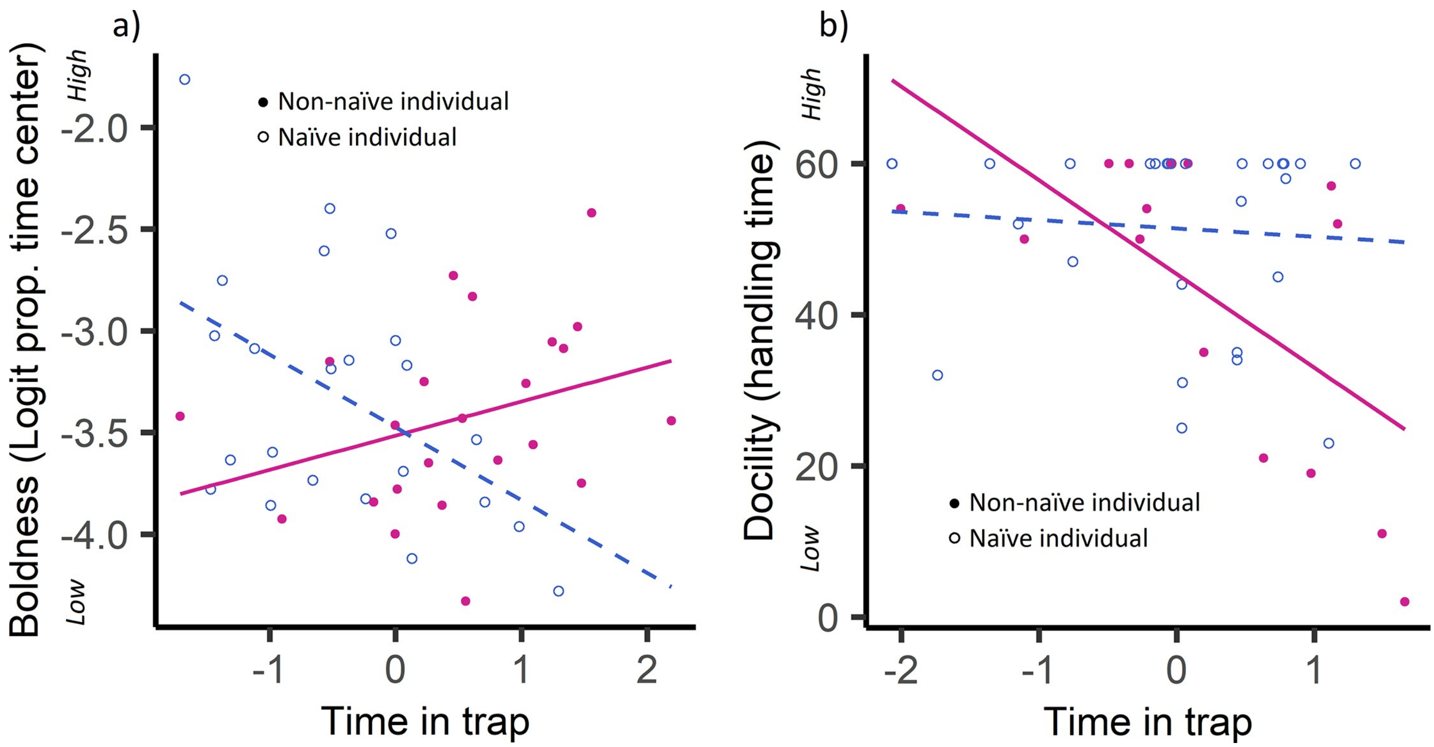

Fig 3. Prior trapping experience influences the behavioral response to trap confinement in deer mice (*Peromyscus maniculatus*) and southern red-backed voles (*Myodes gapperi*). **(a)** Deer mice who were naïve to trapping showed a negative relationship between time in the trap and the proportion of time spent in the center portion of the open-field test. Non-naïve mice showed the reverse relationship. **(b)** Voles who were not naïve to trapping showed a negative relationship between time in the trap and handling time. Plotted are the relationships predicted from the top linear models and raw data points. The variable "time in trap" has been z-standardized, and the variable "prop. time center" is on a logit scale.

https://doi.org/10.1371/journal.pone.0221136.g003

stress responses and the findings have been mixed. It has been shown in southern red-backed voles and meadow voles that live trapping induces an initial stress response, but that this response is not heightened following prolonged confinement inside traps [28,35]. In our study, the observed behavior of red-backed voles in behavioral tests was consistent with these findings and 6 out of 7 behaviors showed no correlation with the time that the animal had spent previously confined inside of a trap. Previous studies investigating the correlation between stress response and duration of trap confinement in deer mice saw that after prolonged time spent in traps, stress-related hormone levels were significantly higher than after a short duration of trap confinement [35]. By contrast, our results show no correlation between 6 out of 7 behavioral measurements and trap confinement duration in the deer mouse.

Although a hormonal change does not necessarily precede a change in behavior, we would expect to see an observable behavioral change in individual deer mice experiencing elevated stress levels (for example, by affecting behaviors that indicate activity level such as speed of locomotion and rearing, or in behaviors indicating stress response such as grooming). Instead, the one behavior in deer mice for which "time in trap" occurred in the top model was the proportion of time spent in the center of the open-field test, a behavior that is most commonly interpreted as indicating the degree of boldness [33,54–58]. Interestingly, our results show that naïve individuals, who had never been trapped previously, behaved more boldly in the open-field test when their confinement duration was short. Non-naïve individuals showed the opposite effect; bolder behavior was seen in animals who had spent longer times in the trap (Fig 3A). This finding suggests that deer mice show some degree of habituation to trapping, and

that their experience during trap confinement is different on their first instance of capture than it is during subsequent captures. While these results are small and difficult to interpret, one possible speculation is that a naïve individual is more stressed by the initial trapping event than by the prolonged confinement in the trap. This would mean that stress levels would be lower after a longer confinement duration than after a short duration, and it is possible that increased stress leads to a proactive coping style [59]. The reverse relationship seen in non-naïve individuals, however, is more difficult to interpret. It is also worth noting that we did not observe an interaction between trap confinement duration and the amount of grooming that non-naïve mice performed in the open-field test which would have been expected since grooming is commonly used to assess anxiety and stress in both a lab and field setting [34,54,60]. Further, we observed high repeatability in the boldness of deer mice, which reinforces the fact that any effect of the habituation to trap confinement on behavior is minimal. In studies where behaviors are only marginally repeatable, it may, however, be especially important to control for test repeat number during analysis to more accurately estimate the proportion of the variance that can be attributed to individual differences [45,61–64].

In voles, the one behavior that was affected by the "time in trap" was handling time. This behavior is commonly used to assess docility [44–46,61]. Our results showed that for non-naïve individuals only, shorter durations in the trap correlated with increased docility (Fig 3B). Similar to our results for deer mice, this response shows a more proactive coping style after prolonged periods of confinement for non-naïve individuals only and may reflect that for an experienced individual, the initial trapping event is less stressful than the period of trap confinement. Again, however, we saw no effect of confinement duration on behaviors that indicate stress and anxiety, and docility was highly repeatable in voles. This strengthens our overall finding that the influence of trap confinement on behavior is minimal.

Since 86% of observed behaviors by deer mice and voles showed no correlation with the variable "time in trap", and all variables commonly used to indicate activity and anxiety showed no correlations, we suspect that the duration of trap confinement is not providing a prolonged stressor for small mammals. It may be noteworthy that the previous trap response studies of deer mice and voles [28,35] used Sherman traps instead of the Longworth traps used in this study. Longworth traps differ from Sherman traps in that they have a separate nest chamber (providing additional warmth and protection) which may help to limit stress. Additionally, we took further steps to minimize stress by ensuring that bedding remained dry (i.e., limiting trapping in adverse weather and replacing damp bedding immediately), and providing ample bait inside the traps. Further, we checked traps twice within a 24-hr period to limit confinement durations (once in the morning, and once just before dark). We cannot speculate about whether these precautions were adequate in our study to stop an increased stress response after the initial stressor of the trapping event, but regardless, prolonged confinement in a Longworth trap does not seem to result in an observable change for the majority of behaviors in either study species.

Future research examining this relationship in other species and other study populations will help to assess and confirm the generalizability of our findings. We suggest future studies quantifying the effects of trap confinement also include data on the physiological stress response, and consider non-repeatable behavioral traits along with personality traits. Furthermore, we suggest that other studies investigating personality in small mammals consider in analyses whether or not animals have been captured previously [61,62]. Finally, the response to stressful situations (as in confinement during live trapping), or an individual's coping style, may itself represent an aspect of an animal's personality [21,59,65]. Within the coping styles framework, it would be interesting to explore to what extent an individual's behavioral response to trap-induced stress might be plastic vs. relatively fixed over time. It is possible that

with a repeated measures design, we could tease apart how much variability exists in the effects of trap confinement on observed behavior and what percent of this variability might be attributable to between individual differences.

Personality studies on wild populations will likely continue to become more common as further research demonstrates the cascade-effects that individual behavioral traits can have on populations and communities [14,16,18,19,66]. Hence, it is critical to ensure that the very process we seek to illuminate is not being confounded by our methods of obtaining data. Our findings provide evidence that time spent inside of Longworth traps does not determine behaviors performed during standardized tests in two different small mammal species. Therefore, our results suggest that personality measurements on wild, trapped small mammals are not regulated by trapping procedures.

Supporting information

S1 Table. Repeatability estimates for target behaviors measured in three behavioral tests (handling bag, emergence, and open-field) in deer mice (*Peromyscus maniculatus*) and southern red-backed voles (*Myodes gapperi*).
(DOCX)

S1 Video. Observed capture event of a southern red-backed vole (*Myodes gapperi*).
(MP4)

S2 Video. Observed capture event of a deer mouse (*Peromyscus maniculatus*).
(MP4)

Acknowledgments

We thank a number of dedicated field and lab technicians for helping with data collection and video analysis, and Bryn Evans for help with map preparation. We thank Malcolm Hunter and two anonymous reviewers for comments on a previous version of this manuscript, and Laura Kenefic (U.S. Forest Service), and Keith Kanoti for maintaining and facilitating research at the Penobscot Experimental Forest. We are especially thankful to Sara Boone for support in the field. Maine Agricultural and Forest Experiment Publication Number 3726.

Author Contributions

Conceptualization: Allison M. Brehm, Sara Tironi, Alessio Mortelliti.

Data curation: Allison M. Brehm, Sara Tironi.

Formal analysis: Allison M. Brehm.

Funding acquisition: Allison M. Brehm, Alessio Mortelliti.

Investigation: Allison M. Brehm, Sara Tironi, Alessio Mortelliti.

Methodology: Sara Tironi, Alessio Mortelliti.

Resources: Alessio Mortelliti.

Writing – original draft: Allison M. Brehm.

Writing – review & editing: Allison M. Brehm, Sara Tironi, Alessio Mortelliti.

References

1. Pennisi E. The power of personality. Science. 2016; 352: 644–647. https://doi.org/10.1126/science.352.6286.644 PMID: 27151848
2. Sih A, Bell AM, Johnson JC, Ziemba RE. Behavioral syndromes: an integrative overview. Q Rev Biol. 2004; 79: 241–277. https://doi.org/10.1086/422893 PMID: 15529965
3. Carere C, Maestripieri D. Animal personalities: behavior, physiology and evolution. Chicago, Illinois: University of Chicago Press; 2013.
4. Gosling SD. From mice to men: What can we learn about personality from animal research? Psychol Bull. 2001; 127: 45–86. https://doi.org/10.1037/0033-2909.127.1.45 PMID: 11271756
5. van Oers K, de Jong G, van Noordwijk AJ, Kempenaers B, Drent PJ. Contribution of genetics to the study of animal personalities: a review of case studies. Behaviour. 2005; 142: 1185–1206. https://doi.org/10.1163/156853905774539364
6. Smith BR, Blumstein DT. Fitness consequences of personality: a meta-analysis. Behavioral Ecology. 2008. pp. 448–455. https://doi.org/10.1093/beheco/arm144
7. Biro PA, Stamps JA. Are animal personality traits linked to life-history productivity? Trends Ecol Evol. 2008; 23: 361–368. https://doi.org/10.1016/j.tree.2008.04.003 PMID: 18501468
8. Dingemanse NJ, Réale D. Natural selection and animal personality. Behaviour. 2005; 142: 1159–1184. https://doi.org/10.1163/156853905774539445
9. Haage M, Maran T, Bergvall UA, Elmhagen B, Angerbjörn A. The influence of spatiotemporal conditions and personality on survival in reintroductions–evolutionary implications. Oecologia. 2017; 183: 45–56. https://doi.org/10.1007/s00442-016-3740-0 PMID: 27722799
10. Dingemanse NJ, Kazem AJN, Re D, Wright J. Behavioural reaction norms: animal personality meets individual plasticity. Trends Ecol Evol. 2009; 25: 81–89. https://doi.org/10.1016/j.tree.2009.07.013 PMID: 19748700
11. Sih A, Kats LB, Maurer EF. Behavioural correlations across situations and the evolution of antipredator behaviour in a sunfish-salamander system. Anim Behav. 2003; 65: 29–44. https://doi.org/10.1006/anbe.2002.2025
12. Wong BBM, Candolin U. Behavioral responses to changing environments. Behav Ecol. 2015; 26: 665–673. https://doi.org/10.1093/beheco/aru183
13. Tuomainen U, Candolin U. Behavioural responses to human-induced environmental change. Biol Rev. 2011; 86: 640–657. https://doi.org/10.1111/j.1469-185X.2010.00164.x PMID: 20977599
14. Sih A, Cote J, Evans M, Fogarty S, Pruitt J. Ecological implications of behavioural syndromes. Ecol Lett. 2012; 15: 278–289. https://doi.org/10.1111/j.1461-0248.2011.01731.x PMID: 22239107
15. Wolf M, Weissing FJ. Animal personalities: consequences for ecology and evolution. Trends Ecol Evol. 2012; 27: 452–461. https://doi.org/10.1016/j.tree.2012.05.001 PMID: 22727728
16. Brehm AM, Mortelliti A, Maynard GA, Zydlewski J. Land-use change and the ecological consequences of personality in small mammals. Ecol Lett. 2019; 22: 1387–1395. https://doi.org/10.1111/ele.13324 PMID: 31207017
17. Miranda AC, Schielzeth H, Sonntag T, Partecke J. Urbanization and its effects on personality traits: a result of microevolution or phenotypic plasticity? Glob Chang Biol. 2013; 19: 2634–2644. https://doi.org/10.1111/gcb.12258 PMID: 23681984
18. Merrick MJ, Koprowski JL. Should we consider individual behavior differences in applied wildlife conservation studies? Biological Conservation. Elsevier Ltd; 2017. pp. 34–44. https://doi.org/10.1016/j.biocon.2017.01.021
19. Lapiedra O, Schoener TW, Leal M, Losos JB, Kolbe JJ. Predator-driven natural selection on risk-taking behavior in anole lizards. Science. 2018; 360: 1017–1020. https://doi.org/10.1126/science.aap9289 PMID: 29853685
20. Berger-Tal O, Blumstein DT, Carroll S, Fisher RN, Mesnick SL, Owen MA, et al. A systematic survey of the integration of animal behavior into conservation. Conserv Biol. 2016; 30: 744–753. https://doi.org/10.1111/cobi.12654 PMID: 26548454
21. Dammhahn M, Almeling L. Is risk taking during foraging a personality trait? A field test for cross-context consistency in boldness. Anim Behav. 2012; 84: 1131–1139. https://doi.org/10.1016/j.anbehav.2012.08.014
22. Carter AJ, English S, Clutton-Brock TH. Cooperative personalities and social niche specialization in female meerkats. J Evol Biol. 2014; 27: 815–825. https://doi.org/10.1111/jeb.12358 PMID: 24666630
23. Bergvall UA, Schäpers A, Kjellander P, Weiss A. Personality and foraging decisions in fallow deer, *Dama dama*. Anim Behav. 2011; 81: 101–112. https://doi.org/10.1016/j.anbehav.2010.09.018

24. Carter AJ, Goldizen AW, Tromp SA. Agamas exhibit behavioral syndromes: bolder males bask and feed more but may suffer higher predation. Behav Ecol. 2010; 21: 655–661. https://doi.org/10.1093/beheco/arq036

25. Ortiz RM, Worthy GAJ. Effects of capture on adrenal steroid and vasopressin concentrations in free-ranging bottlenose dolphins (*Tursiops truncatus*). Comp Biochem Physiol—A Mol Integr Physiol. 2000; 125: 317–324. https://doi.org/10.1016/s1095-6433(00)00158-6 PMID: 10794960

26. Boonstra R, Mccoll CJ, Karels TJ. Reproduction at all costs: the adaptive stress response of male arctic ground squirrels. America (NY). 2009; 82: 1930–1946.

27. Reeder DAM, Kosteczko NS, Kunz TH, Widmaier EP. Changes in baseline and stress-induced glucocorticoid levels during the active period in free-ranging male and female little brown myotis, *Myotis lucifugus* (Chiroptera: Vespertilionidae). Gen Comp Endocrinol. 2004; 136: 260–269. https://doi.org/10.1016/j.ygcen.2003.12.020 PMID: 15028530

28. Fletcher QE, Boonstra R. Impact of live trapping on the stress response of the meadow vole (*Microtus pennsylvanicus*). J Zool. 2006; 270: 473–478. https://doi.org/10.1111/j.1469-7998.2006.00153.x

29. Kenagy NJPGJ. Seasonal changes in plasma testosterone and glucocorticosteroids in free-living male yellow-pine chipmunks and the response to capture and handling. 2000; 245–251.

30. Bosson CO, Islam Z, Boonstra R, Bosson CO, Prés R. The impact of live trapping and trap model on the stress profiles of North American red squirrels. J Zool. 2012; 288: 159–169. https://doi.org/10.1111/j.1469-7998.2012.00941.x

31. Sapolsky RM, Romero LM, Munck AU. How do glucocorticoids influence stress responses? Integrating permissive, suppressive, stimulatory, and rreparative actions*. 2000. Available: https://academic.oup.com/edrv/article-abstract/21/1/55/2423840

32. Carter AJ, Feeney WE, Marshall HH, Cowlishaw G, Heinsohn R. Animal personality: what are behavioural ecologists measuring? Biol Rev. 2013; 88: 465–475. https://doi.org/10.1111/brv.12007 PMID: 23253069

33. Gracceva G, Herde A, Groothuis TGG, Koolhaas JM, Palme R, Eccard JA. Turning shy on a winter's day: effects of season on personality and stress response in *Microtus arvalis*. Ethology. 2014; 120: 753–767. https://doi.org/10.1111/eth.12246

34. Kalueff A V., Stewart AM, Song C, Berridge KC, Graybiel AM, Fentress JC. Neurobiology of rodent self-grooming and its value for translational neuroscience. Nature Reviews Neuroscience. Nature Publishing Group; 2016. pp. 45–59. https://doi.org/10.1038/nrn.2015.8 PMID: 26675822

35. Harper JM, Austad SN. Effect of capture and season on fecal glucocorticoid levels in deer mice (*Peromyscus maniculatus*) and red-backed voles (*Clethrionomys gapperi*). Gen Comp Endocrinol. 2001; 123: 337–344. https://doi.org/10.1006/gcen.2001.7682 PMID: 11589634

36. Benevides AFL, Hansen H, Hess SC. Design and evaluation of a simple signaling device for live traps. J Wildl Manage. 2008; 72: 1434–1436. https://doi.org/10.2193/2006-519

37. Larkin RP, Vandeelen TR, Sabick RM, Gosselink TE, Warner RE. Electronic signaling for prompt removal of an animal from a trap. Wildl Soc Bull. 2003; 31: 392–398. Available: http://www.jstor.org/stable/3784318

38. Brehm AM, Mortelliti A. Mind the trap: large-scale field experiment shows that trappability is not a proxy for personality. Anim Behav. 2018; 142: 101–112. https://doi.org/10.1016/j.anbehav.2018.06.009

39. Brissette JC, Kenefic LS. History of the penobscot experimental forest, 1950–2010. Penobscot experimental forest: 60 years of research and demonstration in Maine, 1950–2010 GTR-NRS-P-123. USDA Forest Service; 2014. pp. 1–20.

40. Brown C, Braithwaite VA. Effects of predation pressure on the cognitive ability of the poeciliid *Brachyraphis episcopi*. Behav Ecol. 2004; 16: 482–487. https://doi.org/10.1093/beheco/ari016

41. Perals D, Griffin AS, Bartomeus I, Sol D. Revisiting the open-field test: what does it really tell us about animal personality? Anim Behav. 2017; 123: 69–79. https://doi.org/10.1016/j.anbehav.2016.10.006

42. Walsh RN, Cummins RA. The open-field test: a critical review. Psychol Bull. 1976.

43. Carere C, Drent PJ, Privitera L, Koolhaas JM, Groothuis TGG. Personalities in great tits, *Parus major*: stability and consistency. Anim Behav. 2005; 70: 795–805. https://doi.org/10.1016/j.anbehav.2005.01.003

44. Boon AK, Réale D, Boutin S. The interaction between personality, offspring fitness and food abundance in North American red squirrels. Ecol Lett. 2007; 10: 1094–1104. https://doi.org/10.1111/j.1461-0248.2007.01106.x PMID: 17877738

45. Montiglio PO, Garant D, Pelletier F, Réale D. Personality differences are related to long-term stress reactivity in a population of wild eastern chipmunks, *Tamias striatus*. Anim Behav. 2012; 84: 1071–1079. https://doi.org/10.1016/j.anbehav.2012.08.010

46. Taylor RW, Boutin S, Humphries MM, Mcadam AG. Selection on female behaviour fluctuates with off-spring environment. J Evol Biol. 2014; 27: 2308–2321. https://doi.org/10.1111/jeb.12495 PMID: 25293495

47. Cleasby IR, Nakagawa S, Schielzeth H. Quantifying the predictability of behaviour: statistical approaches for the study of between-individual variation in the within-individual variance. Methods Ecol Evol. 2015; 6: 27–37. https://doi.org/10.1111/2041-210X.12281

48. Dingemanse NJ, Dochtermann NA. Quantifying individual variation in behaviour: mixed-effect modelling approaches. J Anim Ecol. 2013; 82: 39–54. https://doi.org/10.1111/1365-2656.12013 PMID: 23171297

49. Burnham KP, Anderson DR. Model selection and multimodel inference: a practical information—theoretic approach. 2nd ed. New York: Springer-Verlag New York; 2002.

50. Zuur AF, Leno EN, Walker N, Saveliev AA, Smith GM. Mixed effects models and extensions in ecology with R. New York: Springer-Verlag New York; 2009. https://doi.org/10.1007/978-0-387-87458-6

51. Buckland ST, Burnham KP, Augustin NH. Model selection: an integral part of inference. Biometrics. 2006; 53: 603. https://doi.org/10.2307/2533961

52. R Core Team. R: A language and environment for statistical computing. R Found Stat Comput. 2017. Available: http://www.r-project.org/

53. Bates DM, Maechler M, Bolker BM, Walker S. lme4: Linear mixed-effects models using Eigen and S4. 2015.

54. Choleris E, Thomas AW, Kavaliers M, Prato FS. A detailed ethological analysis of the mouse open field test: effects of diazepam, chlordiazepoxide and an extremely low frequency pulsed magnetic field. Available: www.elsevier.com/locate/neubiorev

55. Eccard JA, Herde A. Consistency in boldness, activity and exploration at different stages of life. BMC Ecol. 2013; 103: 61–68. https://doi.org/10.1016/j.anbehav.2015.02.007

56. Treit D, Fundytus M. Thigmotaxis as a test for anxiolytic activity in rats. Pharmacol Biochem Behav. 1989; 31: 959–962. Available: papers2://publication/uuid/5F4FD48A-712B-4CFC-AC12-0C54750B2FF9

57. Ramos A, Berton O, Mormède P, Chaouloff F. A multiple-test study of anxiety-related behaviours in six inbred rat strains. Behav Brain Res. 1997; 85: 57–69. https://doi.org/10.1016/s0166-4328(96)00164-7 PMID: 9095342

58. Barnett S. The rat—a study in behavior. ANU Press; 1976.

59. Koolhaas JM, de Boer SF, Coppens CM, Buwalda B. Neuroendocrinology of coping styles: towards understanding the biology of individual variation. Front Neuroendocrinol. 2010; 31: 307–321. https://doi.org/10.1016/j.yfrne.2010.04.001 PMID: 20382177

60. Fernández-Teruel A, Estanislau C. Meanings of self-grooming depend on an inverted U-shaped function with aversiveness. Nature Reviews Neuroscience. Nature Publishing Group; 2016. p. 591. https://doi.org/10.1038/nrn.2016.102 PMID: 27466142

61. Martin JGA, Réale D. Temperament, risk assessment and habituation to novelty in eastern chipmunks, *Tamias striatus*. Anim Behav. 2008; 75: 309–318. https://doi.org/10.1016/j.anbehav.2007.05.026

62. Bell AM, Hankison SJ, Laskowski KL. The repeatability of behaviour: a meta-analysis. Anim Behav. 2009; 77: 771–783. https://doi.org/10.1016/j.anbehav.2008.12.022 PMID: 24707058

63. Mazzamuto MV, Cremonesi G, Santicchia F, Preatoni D, Martinoli A, Wauters LA. Rodents in the arena: a critical evaluation of methods measuring personality traits. Ethol Ecol Evol. 2019; 31: 38–58. https://doi.org/10.1080/03949370.2018.1488768

64. Boyer N, Réale D, Marmet J, Pisanu B, Chapuis JL. Personality, space use and tick load in an introduced population of Siberian chipmunks *Tamias sibiricus*. J Anim Ecol. 2010; 79: 538–547. https://doi.org/10.1111/j.1365-2656.2010.01659.x PMID: 20202009

65. Koolhaas JM, Korte SM, De Boer SF, Van Der Vegt BJ, Van Reenen CG, Hopster H, et al. Coping styles in animals: current status in behavior and stress-physiology. Neurosci Biobehav Rev. 1999; 23: 925–935. https://doi.org/10.1016/s0149-7634(99)00026-3 PMID: 10580307

66. Lapiedra O, Chejanovski Z, Kolbe JJ. Urbanization and biological invasion shape animal personalities. Glob Chang Biol. 2017; 23: 592–603. https://doi.org/10.1111/gcb.13395 PMID: 27310334
